# Comparative speed of kill of sarolaner (Simparica™) and afoxolaner (NexGard®) against induced infestations of *Rhipicephalus sanguineus* s.l. on dogs

**DOI:** 10.1186/s13071-016-1375-y

**Published:** 2016-02-19

**Authors:** Robert H. Six, David R. Young, Susan J. Holzmer, Sean P. Mahabir

**Affiliations:** Zoetis, Veterinary Medicine Research and Development, 333 Portage St., Kalamazoo, MI 49007 USA; YVRS, 7243 East Ave, Turlock, CA 95380 USA

**Keywords:** *Rhipicephalus sanguineus* sensu lato, Brown dog tick, Sarolaner, Simparica™, Afoxolaner, Oral, Speed of kill, Isoxazoline

## Abstract

**Background:**

The brown dog tick*, Rhipicephalus sanguineus* sensu lato, commonly infests dogs globally, is the major vector of the pathogen that causes canine monocytic ehrlichiosis and also transmits *Babesia vogeli*. A rapid speed of kill of a parasiticide is essential to reduce the direct deleterious effects of tick infestation and the risk of tick-borne pathogen transmission. The speed of kill of a novel orally administered isoxazoline parasiticide, sarolaner (Simparica™), against *R. sanguineus* sensu lato on dogs was evaluated and compared with afoxolaner (NexGard®) for 5 weeks after a single oral dose.

**Methods:**

Based on pretreatment tick counts, 24 dogs were randomly allocated to oral treatment with either placebo, or label doses of sarolaner (2–4 mg/kg) or afoxolaner (2.5–6.8 mg/kg). Dogs were examined and live ticks counted at 8, 12, and 24 h after treatment and subsequent re-infestations on Days 7, 14, 21, 28, and 35. Efficacy was determined at each time point relative to counts for placebo dogs.

**Results:**

There were no adverse reactions to treatment. Based on geometric means, sarolaner provided >94 % efficacy within 8 h of treatment, and >99 % after 12 and 24 h. Against subsequent weekly re-infestations of ticks, sarolaner achieved ≥91.7 % efficacy (based on geometric means) to Day 35 at 24 h. Sarolaner significantly reduced tick counts versus placebo on Days 0 and 28 at 8 h (*P* ≤ 0.0390), on Days 0 to 14 and 28 at 12 h (*P* ≤ 0.0142), and on all days at 24 h (*P* < 0.0001). By comparison, tick counts for afoxolaner were significantly lower than placebo at 8 h on Days 0 and 28 (*P* ≤ 0.0117), at 12 h on Day 0 only (*P* < 0.0001), and on all days at 24 h (*P* ≤ 0.0078). Significantly more live ticks were recovered from afoxolaner-treated dogs than from sarolaner-treated dogs at 8 and 12 h after treatment (*P* ≤ 0.0286), at 12 h after re-infestation on Days 7 and 28 (*P* ≤ 0.04630), and at 24 h after re-infestations from Day 7 to Day 35 (*P* ≤ 0.0119). At 24 h, efficacy (based on geometric mean counts) of afoxolaner was less than 90 % from Day 7 onwards, and declined to less than 45 % by Day 35, while efficacy for sarolaner was >90 % for 35 days.

**Conclusions:**

In this controlled laboratory evaluation, sarolaner had a faster speed of kill against *R. sanguineus* sensu lato than afoxolaner. The rapid and consistent kill of ticks within 24 h after a single oral dose of sarolaner over 35 days indicates that this treatment will provide highly effective and reliable control of ticks over the entire treatment interval and should reduce the risk of tick-borne pathogen transmission.

## Background

The brown dog tick*, Rhipicephalus sanguineus* sensu lato, is a pest of dogs world-wide [1]. Dogs are the primary host for this tick and all stages develop on the dog though immature stages may also be found on other small mammals [2]. The brown dog tick is unusual in that it is commonly found indoors. Thus, its geographic range is quite extensive as though *R.sanguineus* sensu lato is generally considered to be a tropical tick and relatively cold intolerant, it persists in temperate regions by infesting kennels and homes [2]. Unfed larvae, nymphs and adults can survive for many months off the host but the life cycle can be completed in as little as 2–3 months. When dogs are constantly available as hosts, tick populations can rapidly increase and infestations in kennels or homes can be very difficult to control [3].

*Rhipicephalus sanguineus* sensu lato ticks are vectors of a number of important pathogens globally [1]. The major diseases transmitted by these ticks are canine monocytic ehrlichiosis (caused by *Ehrlichia canis*) and canine babesiosis (caused by *Babesia vogeli*) [4]. The brown dog tick has been shown to harbor *Anaplasma platy*s and *Babesia gibsoni* [5, 6] and is a vector of a number of *Rickettsia* pathogens [1] including the zoonosis Rocky Mountain spotted fever, caused by *Rickettsia rickettsii* [7] and may also be a vector of *Cercopithifilaria* spp. and *Hepatozoon canis* [3].

Tick control and prevention on dogs is important to prevent direct blood loss and irritation caused by the feeding of ticks, and especially to reduce the risk of pathogen transmission. Recently, a new class of systemic compounds, the isoxazolines, have been introduced that have efficacy against ticks and fleas for one month or longer following a single oral dose [8, 9]. These systemically active compounds require the tick to bite in order to kill the parasite. However, the compounds act rapidly to impact the tick’s feeding behavior and cause death of the ticks. One of these, afoxolaner, has been reported to provide >90 % efficacy against *R. sanguineus* sensu lato within 48 h for up to 28 days after a single dose [10, 11], and efficacies of 86.4–99.5 % at 24 h for up to four weeks after treatment [12]. Although product label efficacy claims for ticks are typically based on evaluation at 48 h after treatment or re-infestation [13], the speed of kill is critical in the prevention of feeding and reducing the risk of pathogen transmission which generally requires the tick to attach and feed for 24 to 48 h [14, 15], though recently transmission of *E. canis* has been shown to occur within as little as 3 h after attachment [16].

Sarolaner is a novel isoxazoline which, in a chewable tablet formulation (Simparica™), provides excellent control of fleas and ticks for at least 1 month after a single oral dose (TLMcTier, personal communications). A laboratory study was conducted to determine and compare the speed of kill of sarolaner and afoxolaner (NexGard®) against existing *R. sanguineus* sensu lato infestations and weekly re-infestations on dogs for a period of 5 weeks after treatment with a single dose.

## Methods

### Ethical approval

The study was a masked, negative controlled, randomized laboratory efficacy design conducted in California, USA. Study procedures were in accordance with the World Association for the Advancement of Veterinary Parasitology (WAAVP) guidelines for evaluating the efficacy of parasiticides for the treatment, prevention, and control of flea and tick infestation on dogs and cats [13] and complied with the principles of Good Clinical Practices [17]. The protocol was reviewed and approved by the local Institutional Animal Care and Use Committee. Masking of the study was assured through the separation of functions. All personnel conducting observations or animal care or performing infestations and counts were masked to treatment allocation.

### Animals

Twenty-four, male and female, purpose-bred Beagles ranging in age from 2.5 to 6.5 years and weighing 8.4 to 17.9 kg were used in the study. Each dog was individually identified by a unique ear tattoo and had undergone an adequate wash-out period to ensure that no residual ectoparasiticide efficacy remained from any previous treatment as demonstrated by live tick retention at the host suitability evaluation. Dogs were individually housed in indoor runs such that no physical contact was possible between dogs and they were acclimatized to these conditions for at least 14 days prior to treatment. Dogs were fed an appropriate maintenance ration of a commercial dry canine feed for the duration of the study. Water was available *ad libitum*. All dogs were given a physical exam to ensure that they were in good health at enrollment and suitable for inclusion in the study. General health observations were performed twice daily throughout the study.

### Design

The study followed a randomized complete block design. Dogs were ranked according to decreasing tick counts into blocks of three and within each block a dog was randomly allocated to treatment with either sarolaner, afoxolaner, or placebo. There were eight dogs per treatment group. However, one afoxolaner-treated dog was excluded from efficacy calculations as it was inadvertently underdosed. Dogs were infested with ticks 2 days prior to treatment and then weekly for 5weeks. Tick counts were conducted at 8, 12, and 24 h after treatment and after each subsequent weekly re-infestation.

### Treatment

Day -2 bodyweights were used to determine the appropriate dose to be administered. On Day 0, dogs received either a placebo tablet, the appropriate strength sarolaner chewable tablet (Simparica™), to provide sarolaner at the recommended minimum dose of 2 mg/kg (range 2 to 4 mg/kg), or NexGard® per label directions (afoxolaner at 2.5 to 6.8 mg/kg). All doses were administered by hand pilling to ensure accurate and complete dosing. Each dog was observed for several minutes after dosing for evidence that the dose was swallowed, and also for general health at 1, 4, and 24 h after treatment administration.

### Tick infestation and assessment

The ticks were obtained from a laboratory colony in North Carolina which was initiated in 2008 with locally collected ticks. Engorged females from various locations in the US are introduced annually. Tick infestations were performed on Days -7 (host suitability and allocation), -2, 7, 14, 21, 28, and 35. Prior to each infestation, the dog was lightly sedated with ketamine/xylazine and a precounted aliquot of 50 (±5) viable unfed adult *R. sanguineus* sensu lato were directly applied to the animal. Each dog was examined to remove and count live ticks at 48 h after the initial host suitability infestation. At 8 and 12 (±1) hours after treatment and each subsequent weekly re-infestation, the dogs were examined and live ticks were counted *in situ*; the dogs were examined systematically so that the entire body surface was carefully examined once. At 24 h after treatment and each subsequent weekly re-infestation, the dogs were examined and then thoroughly combed to count and remove live ticks. Each dog was examined for at least 10 min. If ticks were encountered in the last minute, combing was continued in 1 min increments until no ticks were encountered.

### Statistical analysis

The individual dog was the experimental unit and the primary end point was live tick counts. Data for post-treatment live tick counts were summarized with arithmetic (AM) and geometric (GM) means by treatment group and time point. Tick counts were log_e_ (count + 1) transformed prior to analysis in order to stabilize the variance and normalize the data. Using the PROC MIXED procedure (SAS 9.2, Cary NC), transformed counts were analyzed using a mixed linear model. The fixed effects were treatment, time point and the interaction between time point and treatment by time point. The random effects included block, block by treatment interaction and error. Testing was two-sided at the significance level α = 0.05.

The assessment of efficacy for live ticks was based on the percent reduction in the arithmetic and geometric mean live tick counts relative to placebo, as suggested by the most recent guidelines of the WAAVP for systemic acaricides [12] and was calculated using Abbott’s formula:

$$ \%\ \mathrm{reduction}=100 \times \frac{\mathrm{mean}\ \mathrm{count}\ \left(\mathrm{placebo}\right)\hbox{--} \mathrm{mean}\ \mathrm{count}\ \left(\mathrm{treated}\right)}{\mathrm{mean}\ \mathrm{count}\ \left(\mathrm{placebo}\right)} $$

## Results

There were no treatment-related adverse events during the study. Placebo-treated dogs maintained good tick infestations throughout the study with mean tick counts ranging from approximately 25 to 37 (Tables 1, 2 and 3).

**Table 1:** Mean live *Rhipicephalus sanguineus* sensu lato counts and efficacy relative to placebo at 8 hours after treatment and post-treatment re-infestations for dogs treated with a single oral dose of either sarolaner or afoxolaner on Day 0^1^

Treatment		Day of treatment or re-infestation					
		0	7	14	21	28	35
Placebo	Range	19–43	24–39	16–43	21–40	33–42	28–41
	A. mean	32.1	34.1	32.3	32.9	36.5	33.4
	G. mean^2^	31.2^a^	33.8^a^	31.1^a^	32.2^a^	36.4^a^	33.0^a^
Sarolaner	Range	0–15	24–43	19–36	25–42	16–36	26–41
	A. mean	3.5	32.3	27.4	31.0	29.8	32.3
	Efficacy (%)	89.1	5.5	15.1	5.7	18.5	3.4
	G. mean^2^	1.8^b^	31.8^a^	26.8^a^	30.6^a^	29.0^b^	31.9^a^
	Efficacy (%)	94.3	5.8	13.7	5.1	20.2	3.2
	*P*-value vs. placebo	0.0004	0.5773	0.2508	0.6717	0.0390	0.7036
Afoxolaner	Range	3–27	26–48	25–39	26–41	29–34	28–37
	A. mean	11.7	34.4	32.4	33.7	31.4	33.0
	Efficacy (%)	63.5	0.0	0.0	0.0	13.9	1.1
	G. mean^2^	9.0^c^	33.8^a^	32.2^a^	33.4^a^	31.4^b^	32.9^a^
	Efficacy (%)	71.2	0.0	0.0	0.0	13.7	0.4
	*P*-value vs. placebo	0.0070	0.9838	0.7788	0.7690	0.0117	0.9666
	*P*-value vs. sarolaner	0.0238	0.5399	0.0574	0.3106	0.4449	0.6411

^1^ *n* = 7 for afoxolaner, *n* = 8 for placebo and sarolaner groups

^2^Geometric means within columns with the same superscript are not significantly different (*P* > 0.05)

**Table 2:** Mean live *Rhipicephalus sanguineus* sensu lato counts and efficacy relative to placebo at 12 hours after treatment and post-treatment re-infestations for dogs treated with a single oral dose of either sarolaner or afoxolaner on Day 0^1^

Treatment		Day of treatment or re-infestation					
		0	7	14	21	28	35
Placebo	Range	17–42	20–38	15–40	19–40	27–40	25–41
	A. mean	28.6	30.5	30.9	31.9	34.4	32.1
	G. mean^2^	27.5^a^	29.6^a^	29.8^a^	31.1^a^	34.2^a^	31.8^a^
Sarolaner	Range	0–2	8–27	13–25	22–31	15–31	20–38
	A. mean	0.3	20.0	19.8	24.9	24.6	27.3
	Efficacy (%)	99.1	34.4	36.0	22.0	28.4	15.2
	G. mean^2^	0.1^b^	18.9^b^	19.1^b^	24.7^a^	24.2^b^	26.7^a^
	Efficacy (%)	99.5	36.1	35.9	20.5	29.2	15.9
	*P*-value vs. placebo	<0.0001	0.0142	0.0061	0.0754	0.0020	0.0715
Afoxolaner	Range	0–5	26–42	16–34	23–36	25–33	23–35
	A. mean	2.4	34.4	26.3	29.9	29.9	30.7
	Efficacy (%)	91.5	0.0	14.9	6.3	13.1	4.4
	G. mean^2^	1.7^c^	33.9^a^	25.5^a,b^	29.4^a^	29.7^a^	30.5^a^
	Efficacy (%)	93.8	0.0	14.6	5.5	13.0	4.1
	*P*-value vs. placebo	<0.0001	0.3251	0.3213	0.6858	0.0553	0.6435
	*P*-value vs. sarolaner	0.0286	0.0015	0.0732	0.0770	0.0463	0.1679

^1^ *n* = 7 for afoxolaner, *n* = 8 for placebo and sarolaner groups

^2^Geometric means within columns with the same superscript are not significantly different (*P* > 0.05)

**Table 3:** Mean live *Rhipicephalus sanguineus* sensu lato counts and efficacy relative to placebo at 24 hours after treatment and post-treatment re-infestations for dogs treated with a single oral dose of either sarolaner or afoxolaner on Day 0^1^

Treatment		Day of treatment or re-infestation					
		0	7	14	21	28	35
Placebo	Range	19–43	17–41	11–34	20–39	24–38	19–37
	A. mean	28.8	30.5	26.9	30.0	31.5	28.0
	G. mean^2^	27.8^a^	29.5^a^	25.6^a^	29.2^a^	31.2^a^	27.3^a^
Sarolaner	Range	0–1	0–2	0–3	0–1	0–6	0–11
	A. mean	0.1	0.6	1.5	0.3	1.3	3.4
	Efficacy (%)	99.6	98.0	94.4	99.2	96.0	87.9
	G. mean^2^	0.1^b^	0.5^b^	1.1^b^	0.2^b^	0.7^b^	2.3^b^
	Efficacy (%)	99.7	98.3	95.7	99.4	97.8	91.7
	*P*-value vs. placebo	<0.0001	<0.0001	<0.0001	<0.0001	<0.0001	0.0001
Afoxolaner	Range	0–0	0–11	2–16	0–15	1–22	11–31
	A. mean	0.0	4.1	7.6	8.7	9.6	16.0
	Efficacy (%)	100.0	86.4	71.8	71.0	69.6	42.9
	G. mean^2^	0.0^b^	3.1^c^	6.2^c^	6.2^c^	7.3^c^	15.2^c^
	Efficacy (%)	100	89.4	76.0	78.8	76.5	44.5
	*P*-value vs. placebo	<0.0001	0.0002	0.0006	0.0078	0.0041	0.0021
	*P*-value vs. sarolaner	0.3833	0.0119	0.0028	0.0022	0.0022	0.0008

^1^ *n* = 7 for afoxolaner, *n* = 8 for placebo and sarolaner groups

^2^Geometric means within columns with the same superscript are not significantly different (*P* > 0.05)

At the 8-hour time point, treatment with sarolaner resulted in significantly lower GM tick counts than placebo-treated dogs (*P* ≤ 0.0390) on Days 0 and 28, and efficacy (GM) was 94.3 and 20.2 %, respectively (Table 1). Treatment with afoxolaner resulted in significantly lower tick counts than placebo at 8 h on Days 0 and 28 as well (*P* ≤ 0.0117), with efficacy (GM) of 71.2 and 13.7 %, respectively (Table 1). Sarolaner had superior efficacy than afoxolaner at 8 h against the existing infestation (*P* = 0.0238), but there were no significant differences between the GM mean tick counts at 8 h for sarolaner and afoxolaner-treated dogs on any day for the subsequent post-treatment re-infestations (*P* ≥ 0.0574).

At the 12-hour time point, sarolaner-treated dogs had significantly lower tick counts than placebo-treated dogs (*P* ≤ 0.0142) from treatment through Day 14 and on Day 28, with efficacy (GM) ranging from 29.2 to 99.5 % (Table 2). Treatment with afoxolaner resulted in significantly lower tick counts than placebo at 12 h on Day 0 only (*P* < 0.0001) with efficacy (GM) of 93.8 %. Efficacy for afoxolaner was ≤14.6 % on all other days (Table 2). Tick counts were significantly higher for afoxolaner-treated dogs than for sarolaner-treated dogs on Days 0, 7, and 28 (*P* ≤ 0.0463).

**Fig. 1:**
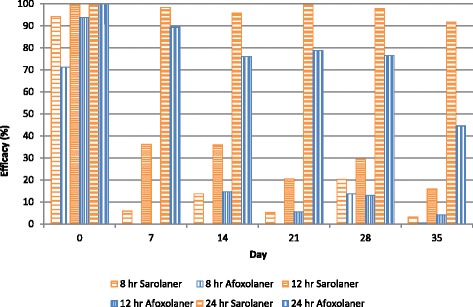
Percent efficacy based on geometric mean counts relative to placebo at 8, 12, and 24 hours after treatment and post-treatment re-infestations of *Rhipicephalus sanguineus* sensu lato for dogs treated with a single oral dose of either sarolaner or afoxolaner on Day 0

At the 24-hour time point, both treatments resulted in significantly lower tick counts than placebo-treated dogs (*P* ≤ 0.0078) throughout the study, and sarolaner-treated dogs also had significantly fewer ticks than afoxolaner-treated dogs (*P* ≤ 0.0119) following all post-treatment re-infestations (Days 7 to 35, Table 3). Treatment with sarolaner resulted in efficacy (GM) of at least 91.7 % through Day 35, while efficacy (GM) for dogs treated with afoxolaner declined below 90 % from Day 7 onwards (Table 3, Fig. 1).

## Discussion

A single dose of sarolaner resulted in the rapid reduction in *R.sanguineus* sensu lato ticks that had been applied two days previously and in the rapid kill of re-infestations for a full month after treatment. Efficacy of ≥91.7 % (based on GM) was achieved within 24 h for 35 days. This consistent efficacy at 24 h after treatment and subsequent re-infestations for 35 days was significantly superior to that of afoxolaner for all post-treatment re-infestations. The decline in efficacy against *R. sanguineus* sensu lato for afoxolaner from 89.4 % on Day 7 to 44.5 % by Day 35 after a single treatment can be compared with published information. Kunkle *et al.* [10] reported that a single oral dose of afoxolaner resulted in efficacies (based on GM) against *R. san*g*uineus* sensu lato of 98.5 % and 100 % at 48 h after treatment and ranging from 98.1 to 99.4 % for subsequent weekly re-infestations to Day 35, but did not assess efficacy at earlier time points. Another study evaluated the efficacy of afoxolaner at 24 h time points [12], but the first evaluation (AM efficacy of 99.5 %) was not conducted until after the second treatment when ticks were applied immediately after the dogs were dosed; efficacy (AM) of afoxolaner at 24 h for ticks re-infested 21 days after the second treatment was 93.7 %, and at 28 days after the third monthly treatment efficacy (AM) was 86.4 %. As some cumulative effect of repeat dosing at 28 day intervals would be expected, these published data are in good agreement with the 24 h efficacy seen in the current study following a single dose (e.g. AM efficacy for afoxolaner of 71.0 and 69.6 % on Days 21 and 28, respectively after a single dose). The 24 h AM efficacy for sarolaner on these two days was 99.2 and 96.0 %, respectively.

The rapid kill of ticks is critical to reduce the risk of tick-borne pathogen transmission and to alleviate the irritation and blood loss that is a direct consequence of tick feeding. Thus, the speed of kill of sarolaner against *R. sanguineus* sensu lato and its consistent high efficacy over the full month following a single oral dose should provide a marked reduction in the discomfort caused by tick infestation and also reduce the risk of a treated dog becoming infected with the pathogens transmitted by *R. sanguineus* sensu lato.

## Conclusions

This study confirmed the excellent acaricidal efficacy of sarolaner against *R. sanguineus* sensu lato after a single oral administration, and demonstrated that ticks were killed rapidly with the vast majority controlled within 24 h for 35 days. Efficacy for sarolaner was higher than that of afoxolaner at 8 and 12 h after treatment, and consistently superior against re-infestations from Day 7 onwards at 24 h. Thus, sarolaner (Simparica™) offers the pet owner and veterinarian a highly effective oral product with a rapid speed of kill against ticks for the entire month following a single oral dose. It provides a new option for the treatment and prevention of tick infestation that should also reduce the risk of tick-borne pathogen transmission.
